# Pine Bark as a Lignocellulosic Resource for Polyurethane Production: An Evaluation

**DOI:** 10.3390/polym18010096

**Published:** 2025-12-29

**Authors:** Alexander Arshanitsa, Matiss Pals, Alexandra Vjalikova, Laima Vevere, Oskars Bikovens, Lilija Jashina

**Affiliations:** Latvian State Institute of Wood Chemistry, Dzerbenes Street 27, LV-1006 Riga, Latvia; matiss.pals@kki.lv (M.P.); aleksandravjalikova@gmail.com (A.V.); laima.vevere@kki.lv (L.V.); oskars.bikovens@kki.lv (O.B.); lilija_jasina@inbox.lv (L.J.)

**Keywords:** pine bark, extractives, propylene carbonate, oxypropylation, polyurethane, filler, cone calorimeter

## Abstract

This study explores the potential of pine bark—a highly accessible and underexploited by-product of forestry and food processing—as a renewable raw material for rigid polyurethane (PUR) foam production. Under optimal extraction conditions, water-soluble extractives rich in carbohydrates were isolated from biomass with a yield of 25% and subsequently condensed with propylene carbonate (PC) to produce bio-based polyols. The polyols synthesized at a PC/OH molar ratio ranging from 1 to 5 were incorporated into rigid PUR foam formulations as substitutes for commercial polyether polyols. The foams containing bio-polyols synthesized at a PC/OH ratio of 3 demonstrated the highest compressive strength and thermal insulation performance, exceeding those of the reference material by 30% and 9%, respectively, and exhibited enhanced thermo-oxidative stability. Incorporation of extracted bark up to 10 wt% as a filler in the PUR matrix led to a decrease in mechanical properties to the level of the reference foam and a 19% reduction in thermal insulation capacity, without affecting the closed-cell content. Cone calorimetry revealed that both filled and unfilled bio-polyol-based PUR foams exhibited lower degradation rate, heat release rate, and total smoke release compared with the reference material, indicating reduced flammability and a lower tendency toward fire propagation.

## 1. Introduction

Synthetic plastics are an essential part of modern human society, with a global annual production of around 390.7 MT in 2021 [1], and production is expected to triple by 2060 [2]. However, more than 97% of plastics are currently derived from petroleum resources, which creates significant environmental challenges [3].

In 2018, the European Union (EU) adopted the European Strategy for Plastics as a constituent part of the EU’s Circular Economy Action Plan [1]. The main objective of this strategy is to protect the environment by reducing greenhouse gas emissions through a decrease in dependence on fossil raw materials. Two major directions of the strategy are the substitution of fossil precursors for plastic production with renewable alternatives and the increased recycling of plastic materials. In this context, the development of new and/or greener polymer materials with higher performance and lower environmental impact than their traditional fossil-based equivalents is a priority.

Polyurethanes (PUs) are among the most versatile classes of polymers. Owing to their outstanding properties and the possibility of tailoring their characteristics, they are widely used in numerous industrial and everyday applications [4]. The global production volume of PU is projected to reach 29.3 million tons by 2030 [2]. The major pathway for PU production on a commercial scale is the stoichiometric condensation of polyols with isocyanates, both predominantly derived from fossil-based precursors [5]. Isocyanates are highly toxic and carcinogenic compounds and, currently, no green alternative exists for their commercial use [5]. A highly promising approach in this regard is the synthesis of more sustainable, isocyanate-free polyurethanes (NIPUs) through the reaction of cyclic carbonates with diamines, which yields hydroxyl-containing polyurethanes [6,7]. It is important to note that this method is still under investigation in laboratories, and its commercialization remains at an early stage. Therefore, the substitution of fossil-based polyols with renewable alternatives in conventional PU systems is currently recognized as the most viable pathway for developing bio-based polyurethane plastics [8,9].

Lignocellulosic biomass, particularly forestry and wood-processing waste, has been recognized as a highly accessible and low-cost resource for the development of renewable polyols. Its composition—comprising lignin, carbohydrates, and extractives containing hydroxyl groups available for modification—enables the transformation of solid biomass into liquid polyols that are highly reactive with isocyanates [10,11].

Tree bark is an underutilized lignocellulosic resource, with a global annual production estimated at 300–400 million m^3^. In Europe, about 69% of this comes from coniferous species, mainly pine, spruce, and fir. This renewable resource is potentially suitable for high-value applications, including PU development, beyond its traditional uses in combustion, composting, and garden mulch production [12,13].

The general method for transforming tree bark into liquid polyols is oxyalkylation with propylene oxide in alkaline media, similar to the oxyalkylation of various lignins developed by Glasser [14,15,16].

The carcinogenicity of propylene oxide (PO), along with its high flammability and low explosive limit in air, are major disadvantages of this method [17].

Unlike other types of lignocellulosic biomass, tree bark has a high content of extractives, consisting predominantly of low-molecular-weight non-lignin polyphenolics and carbohydrates, which makes it particularly feasible for polyol production. Unlike primary metabolites, hydrophilic extractives can be isolated from bark using environmentally friendly extraction methods with water as the solvent [18,19].

In earlier work, liquid polyols suitable for polyurethane processing were synthesized by the oxypropylation of isolated black alder bark extractives using propylene carbonate (PC) instead of hazardous PO [20]. This process is non-flammable, operates under atmospheric pressure and at lower temperatures, and employs non-hazardous, biodegradable PC. It is recognized as a green alternative to conventional oxypropylation [21].

In this work, pressurized-water extraction of pine bark in a Parr reactor was studied with a focus on the yield of carbohydrate-enriched extractives. The isolated extractives were subsequently transformed into liquid polyols by oxypropylation with PC at different PC/OH molar ratios in the presence of catalyst 1,8-diazobycyclo[5.4.0]undec-7 ene (DBU). This catalyst approves its efficiency in comparison with alkali catalysts in oxypropylation of lignin, black alder bark extractives enriched with diarylheptanoids [20,21,22,23]. The resulting bark-extractive-derived polyols were tested in rigid polyurethane (PUR) foam formulations. This type of engineering material, the most in-demand among all types of foam—representing 26% of the global PU market—is widely used across various industries due to its unique combination of low density, high thermal insulation, and favorable mechanical properties [24].

The mechanical and morphological properties, thermal stability, and combustion behavior of PUR foams based on bio-polyols were studied and compared with those based on commercial polyols. Due to the orientation of the modern polyurethane industry toward environmentally friendly processing and the development of bio-plastics, scientific interest in biofillers derived from renewable raw materials has increased significantly [25,26,27]. Therefore, the effect of the residual bark remaining after extraction, used as a natural filler in bio-polyol-based PUR foam composition, on its properties was also evaluated.

## 2. Materials and Methods

### 2.1. Biomass and Chemicals

Bark was separated by manual debarking from approximately 57-year-old pine trees (*Pinus sylvestris*) harvested in the Limbaži municipality of Latvia.

Propylene carbonate (PC) and the catalyst 1,8-diazabicyclo [5.4.0]undec-7-ene (DBU), used for the oxypropylation of pine bark extractives, were supplied by Merck (Darmstadt, Germany).

Commercial polyols used in the reference polyurethane (PUR) foam formulations included glycerol-based trifunctional polyether polyol Lupranol 3300 (OHV = 400 mg KOH∙g^−1^, viscosity = 0.323 Pa∙s) and sorbitol-based hexafunctional polyether polyol Lupranol 3422 (OHV = 490 mg KOH∙g^−1^, viscosity = 22.8 Pa∙s), both supplied by BASF (Ludwigshafen, Germany). Polymeric diphenylmethane diisocyanate (pMDI), with an [NCO] content of 7.5 mmol∙g^−1^ and an average functionality of 2.7, was also sourced from BASF.

Additional components used in the formulation of both reference and bark biomass-containing PUR foams included the amine catalyst Polycat 5 (Evonik, Essen, Germany), the physical blowing agent Opteon™ 1100 (Chemours, Wilmington, DE, USA), and the silicone-based surfactant Niax^®^ L-6915 (Momentive Performance Materials Inc., Leverkusen, Germany). Tris(1-chloro-2-propyl) phosphate (TCPP) was used as a plasticizer and flame retardant (Albermarle, Louvain-la-Neuve, Belgium). The residual extracted pine bark was incorporated into the PUR foam formulations containing oxypropylated bark extractives, serving as a natural filler.

### 2.2. Water Extraction of Pine Bark

Before extraction, pine bark was air-dried to a moisture content of approximately 10% and then ground using a knife-type mill (Retsch 100, Retsch, Düsseldorf, Germany) equipped with a 2 mm sieve. A 100 g portion of the resulting powder was loaded into a 1 L Parr reactor equipped with an oil-heated jacket. Subsequently, 590 mL of deionized water was added. The reactor was purged with argon for 5 min, sealed, and then heated at an average rate of 10 °C∙min^−1^ to the desired temperature within the range of 100–200 °C and excess pressure of 1–15 bar under continuous stirring.

Extraction was conducted under isothermal conditions for 0–1.5 h, with continuous stirring at 150 rpm, followed by deactivation of heating and cooling to 50 °C. After unloading, the suspension was allowed to cool to room temperature. The liquid fraction, enriched with extractives, was separated by filtration using a Büchner funnel, followed by lyophilization. The solid residue was air-dried. The powder-like extractives were stored in tightly sealed plastic containers and kept in a refrigerator at –17 °C.

### 2.3. Characterization of Pine Bark and Isolated Extractives

Elemental analysis (C, H, N) of untreated bark was performed using a Vario MACRO elemental analyzer (ELEMENTAR Analysensysteme Gmbh, Langensebold, Germany), and ash content was determined as a residue after ignition at 550 ± 5 °C a Carbolite ELF 11/6 B furnace (Carbolite Gero, Hope, Derbyshire, UK).

The total monomeric sugar content in extractives with complete hydrolysis was determined by the alditol acetate method with GC-FID quantification according to [28] using 1-O-Methyl-D-glucose as an internal standard. Agilent 6850 series GC (Santa Clara, CA, USA) instrument with 30 m DB-1701 column was used. Glucose, galactose, mannose, xylose, arabinose, and rhamnose were used for calibration. All chemicals used for analysis were purchased from Merck (Darmstadt, Germany).

UHPLC-ESI-MS/MS analysis. Sample analysis was performed using an Acquity UPLC system (Waters Corp., Singapore) equipped with a BEH C18 UPLC column (2.1 × 50 mm, 1.7 µm). Separation was carried out at a flow rate of 0.35 mL·min^−1^ using a 0.1% aqueous solution of formic acid and acetonitrile as the mobile phases. The injection volume was 2 µL. The detection conditions are described in detail elsewhere [29].

The total polyphenolic content (TPC) in the extractives was determined using the Folin–Ciocalteu method as described in [30].

Analytical pyrolysis (Py-GC/MS/FID analysis) of biomass was performed using a Micro Double-shot Pyrolyzer Py-3030D (Frontier Laboratories Ltd., Fukushima, Japan) directly coupled with the Shimadzu GC/MS/FID-QP ULTRA 2010 apparatus (Kyoto, Japan), as described in detail in [29].

FTIR spectra of biomass and cryogenic ground PUR foams was performed by the KBr method, using a Thermo Scientific Nicolet iS550 spectrometer (Norristown, PA, USA). The analysis was conducted in the range of 4000–400 cm^−1^ at a scan resolution and number of scans 4 cm^−1^ and 32 scans, respectively

### 2.4. Oxypropylation of Pine Bark Extractives

Oxypropylation of pine bark extractives with PC was performed at 150 °C and atmospheric pressure according to the methodology developed earlier by the authors [20]. The amount of PC in the reaction mixture was adjusted to achieve molar PC/OH ratios in the range of 1–5, while maintaining a constant molar DBU/OH ratio of 0.05. The progress of PC conversion in the polyols was monitored by FTIR after 1, 2, 3, 4, 5, and 24 h of reaction. If complete conversion was not achieved, monitoring was continued at 2 h intervals. Complete PC conversion was confirmed by the disappearance of the characteristic absorption band of PC at ~1870 cm^−1^ in the FTIR spectra of the products.

### 2.5. Characterization of Polyols

The total content of OH groups in extractives and polyols was measured by acetylation method with acetic anhydride. The free acetic acid formed from unreacted anhydride is quantified by potentiometric titration of aliquot with 0.1 N NaOH [31].

The acid value was determined according to the ISO 2114:2000 standard.

FTIR spectra analysis of bio-polyols was performed using the ATR technique. A Thermo Scientific Nicolet iS550 spectrometer (Norrison, PA, USA) equipped with an ATR ZnSe and diamond crystals top plate was used. The analysis was performed in the range of 4000–400 cm^−1^ at a scan resolution and number of scans of −4 cm^−1^ and 32 scans, respectively.

The water content in bio-polyols was determined by Karl Fisher titration using an automatic titrator Model 275 KF (Denver Instrument, Bohemia, NY, USA).

The rheological tests were performed at 25 °C using the Anton Paar Modular Compact Rheometer MCR 92 (Anton Paar, Graz, Austria) with a cone-plate measuring system. The standard flow curve was constructed in the range of shear rates from 0.1 to 1000 s^−1^. Apparent viscosity of polyols at a shear rate of 50 s^−1^ was detected.

### 2.6. PUR Foam Preparation

The free-rise PUR foams were prepared using two component systems consisting of polyol and isocyanate components. The amount of PMDI in formulations was calculated taking into account equivalent weights of used polyols, water, and isocyanate to achieve a molar NCO/OH ratio equal to 1.15.

The method of formulation calculation, as well as the sequence of steps and the conditions for obtaining PUR foams, are described in detail in our previous publication [32].

Approximately 40 g of polyol containing the specified amounts of catalyst (Polycat 5), surfactant (Niax Silicone, Momentive, Leverkusen, Germany), and flame retardant (TCPP) were thoroughly mixed at 3000 rpm for 1 min in a 1 L paperboard cup using a high-speed mechanical stirrer. Subsequently, the physical blowing agent (Opteon™ 1100, Chemours, Wilmington, DE, USA) was added and premixed at 3000 rpm for 15 s under the same conditions. Subsequently, pMDI was added, and the mixture was stirred for 10 s. For filled compositions, the required amount of filler was first dispersed in the polyol, followed by the addition of the other components as described above. Foaming was carried out in the same cup. Each PUR foam formulation was prepared in duplicate.

### 2.7. PUR Foam Characteristics

The foaming dynamic was studied using a Qualification System FOAMAT 285 [33].

To prepare samples for FTIR and TG analysis the PUR foams were ground into a powder-like material using a ball mill from Retch GmbH CryoMill at the temperature of liquid nitrogen (−196 °C), as described in [32].

Apparent density was measured according to ISO 845:2006.

The closed-cell content by volume was measured using a helium pycnometer AccuPyc II 1340 (Micrometrics, Norcross, GA, USA) according to ISO 4590:2016.

The compressive strength and modulus of PUR foams in the foaming direction were determined according to ISO 844:2021 using Zwick/Roell Z100 universal testing machine (Zwick Roell, Ulm, Germany). Six cubic samples, 30 mm in size, were tested for each PUR foam composition Based on stress–strain curves, obtained compression strength and Young’s modulus were automatically calculated using Zwick/Roell Z100 software (testXpert II) (Figure S10).

Non-isothermal TG/DTG thermal analysis of initial and extracted bark, as well as cryogenically ground PUR foam samples (~20 mg), was performed under argon and in air using a Setaram Setline device (Setaram, Caluire-et-Cuire, France). A 100 µL corundum crucible was used for this purpose. The temperature was ramped from 25 °C to 700 °C at a heating rate of 5 °C·min^−1^. Data processing was carried out using Calisto 2.0 software. Three replicates were performed for each composition.

Air-dried residual pine bark was ground in a Fritsch Pulverisette 5/2 planetary mill (Berlin, Germany) for 1 h and then oven-dried at 100 °C for 24 h prior to use as a natural filler.

The thermal conductivity coefficient (λ) was measured by a FOX 200 heat flow meter (TA Instruments, New Castle, DE, USA) at an average temperature of 10 °C (cold plate: 0 °C, and hot plate: +20 °C, sample dimensions: 200 × 200 × 40 mm), following the ISO 8301:1991 standard.

A cone calorimeter test was conducted to evaluate the combustion properties of PUR foam compositions using an FTT Dual Cone Calorimeter in accordance with ISO 5660-1:2015. PUR foam specimens with dimensions of 100 × 100 × 24 mm were exposed to a constant heat flux of 35 kW/m^2^ to achieve ignition. The test duration was 300 s, and each composition was tested in triplicate

## 3. Results and Discussion

### 3.1. Characterization of Pine Bark

**Figure 1:**
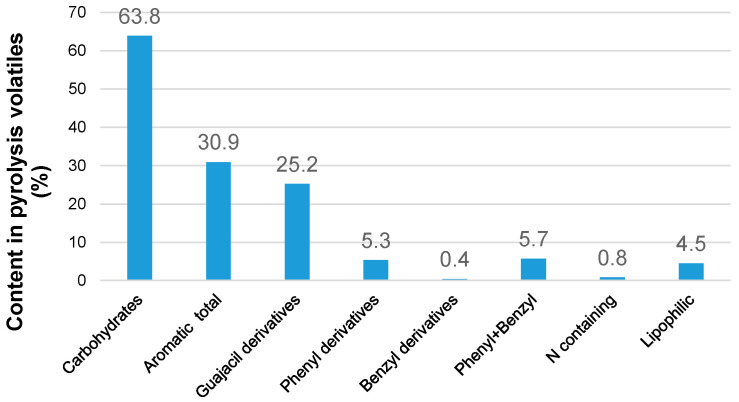
Relative contents of different constituent-derived products in the pyrolysis volatiles of pine bark biomass, based on Py-GC/MS data normalized to 100%.

Analytical pyrolysis combined with gas chromatography/mass spectrometry (Py-GC/MS) was used to evaluate the relative contents of the main groups of constituents, including aromatics, carbohydrates, lipophilic compounds, and nitrogen-containing compounds in pine bark (Figure 1).

The results indicate that carbohydrate-derived products, originating from both cellulose and hemicellulose, constituted the predominant fraction of the pyrolysis volatiles. These were followed by aromatic compounds, primarily methoxylated phenolics derived from lignin. Approximately 20% of the total aromatic fraction consisted of phenyl derivatives, which are likely to originate from both lignin and polyphenolic extractives.

The elemental composition and ash content of the sample under study are typical of softwood bark [34]. The lignin content, which includes both the acid-insoluble (Klason lignin) and acid-soluble fractions, was close to the proportion of aromatics detected by Py-GC/MS (Table 1). The yield of extractives obtained by Soxhlet extraction with non-polar hexane (ε = 1.88) was approximately four times lower than that obtained with comparatively polar 96% ethanol (dielectric constant ε ≈ 27). These results are consistent with the low proportion of non-polar lipophilic compounds in pine bark, as revealed by Py-GC/MS (Table 1). Based on these findings, it can be proposed that ‘green’ extraction with highly polar water at elevated temperatures may provide comparatively high yields of hydrophilic extractives containing hydroxyl groups.

### 3.2. Effect of Extraction Regimes on the Yield of Extractives and Their Composition

**Figure 2:**
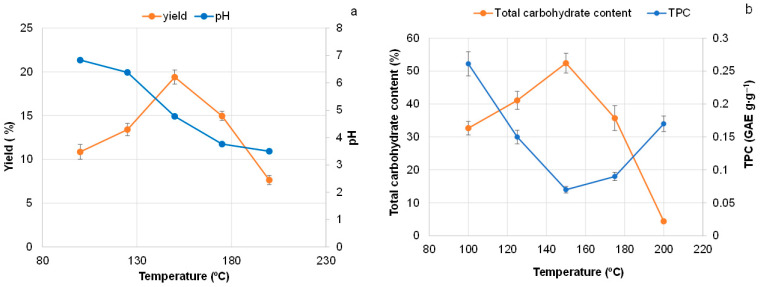
Effect of isothermal extraction temperature on (**a**) pine bark extractive yield and liquid fraction pH, and (**b**) total polyphenolic (TPC) and monomeric carbohydrate contents.

Taking into account the principles of “green” extraction, the isolation of extractives was performed using deionized water as a solvent without any additives [35]. It can be considered that one-step extraction in a PARR reactor is highly suitable for the upscaling of pine bark extraction. This process can be implemented on an industrial scale using a simple reactor equipped with a heating jacket, an agitator, a manifold for evacuation and inert gas supply, a Nutsch filter, and the capability to operate at an excess pressure of 5 bar. The main motivation for choosing this extraction method, instead of more modern but less scalable techniques—such as accelerated solvent extraction or microwave extraction conducted previously [29]—was scalability and simplicity. The temperature of extraction was varied in the range of 100–200 °C with the focus on finding the highest yield of extractives (Figure 2).

As shown, the increase in extraction temperature is accompanied by a steady decrease in the acidity of the solution (Figure 2a). This can be explained by the deacetylation of hemicellulose, predominantly xylan, resulting in the liberation of acetic acid, which promotes hemicellulose depolymerization through autohydrolysis [36].

The yield of extractives does not increase steadily with rising extraction temperature, but reaches a maximum at 150 °C.

It is known that polyphenolics and carbohydrates are the dominant components of hydrophilic pine extractives. Polyphenolics are represented mainly by condensed tannins consisting of proanthocyanidins (PACs), the oligomeric forms of catechin, with admixtures of lignans [37].

The carbohydrate fraction consists of mono- and oligosaccharides derived from hemicelluloses, such as galactoglucomannans and arabinomethylglucuronoxylans [12,37]. In addition to free carbohydrates, bound carbohydrates in the form of phenolic glucosides were also detected in pine bark extractives. Notably, compared with the hot-water extracts of tropical species, European softwood bark extracts contain a lower proportion of polyphenolic compounds and a higher proportion of carbohydrates [37].

In this study, it was shown that extractives obtained at the lowest temperature (100 °C) contained the highest proportion of polyphenolic (TPC = 0.261 GAE g∙g^−1^). The lowest polyphenolic content (TPC = 0.08 GAE·g^−1^) was detected in the fraction isolated at 150 °C, which was the most enriched in carbohydrates (Figure 2).

Using UHPLC, along with the predominantly present carbohydrates, the main phenolic compounds identified in pine bark water extractives isolated at 150 °C include epicatechin, rutin, quercetin, and proanthocyanidin dimers of A and B type (Figure S1, Table S1). With increasing extraction temperature up to 150 °C, the TPC of the extractives steadily decreased, whereas the carbohydrate content increased, reaching up to 50% of the total extractives weight. This corresponded to the maximum yield of extractives (Figure 2a,b, Table S2). This observation can be explained by the fact that polyphenolics were extracted almost completely at 100 °C, while the fraction of dissolved carbohydrates increased with rising temperature. As a result, the relative polyphenolic content decreased due to the dilution effect, whereas the overall yield of extractives increased (Figure 2a).

The further increase of extraction temperature to 175 and 200 °C significantly decreases the total yield of extractives and the carbohydrate content, resulting in a twofold increase of TPC compared with its minimum value (Figure 2b, Table S2). This can be explained by the dehydration of carbohydrates promoted by the hydrothermal treatment of biomass in an acidic medium at temperatures above 150 °C, leading to the formation of furfural and 5-hydroxymethylfurfural (HMF) [38,39].

**Figure 3:**
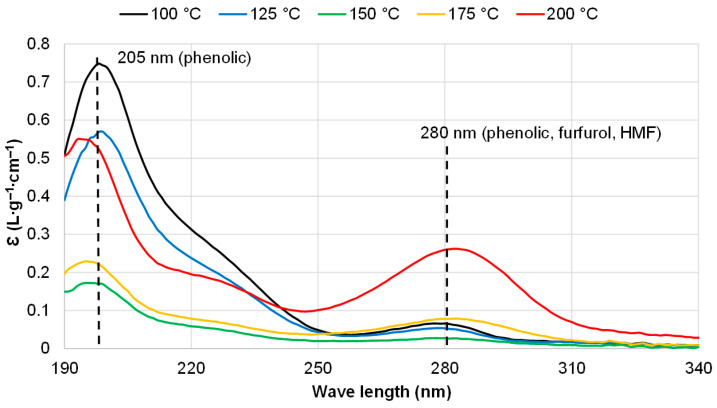
The UV spectra of liquid fractions obtained by the water extraction of pine bark at different temperatures.

This observation was confirmed by the UV spectra of the liquid fractions isolated after pine bark extraction at different temperatures (Figure 3). It is known that both furfural and HMF exhibit strong absorption around 280 nm, corresponding to the n → π* transition of the conjugated carbonyl group, along with phenolic compounds [40]. Absorption at approximately 205 nm is attributed to all aromatic compounds, including phenolic and polyphenolic compounds, with an extinction coefficient about ten times higher than that at 280 nm [41].

**Figure 4:**
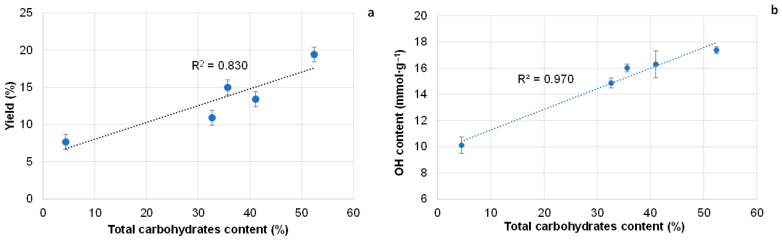
Effect of total carbohydrate content in extractives on their yield (**a**) and on the OH group content in them (**b**).

The highest absorption intensity at 280 nm was observed for the liquid fraction obtained after pine bark extraction at 200 °C, due to the highest content of furfural and 5-HMF. In contrast, the highest absorption intensity at 205 nm for the fraction obtained at 100 °C indicates the largest portion of polyphenolics, which corresponds to the TPC values of the corresponding extractives. The liquid fraction isolated at 150 °C exhibited minimal absorption intensities at both 205 and 280 nm, reflecting the absence of furan derivatives, the lowest content of phenolics, and, correspondingly, the highest carbohydrate content—ultimately resulting in the highest yield of extractives (Figure 4a).

As carbohydrates are the dominant compounds in water extracts of pine bark, avoiding their dehydration is necessary to achieve a high yield of extractives.

**Figure 5:**
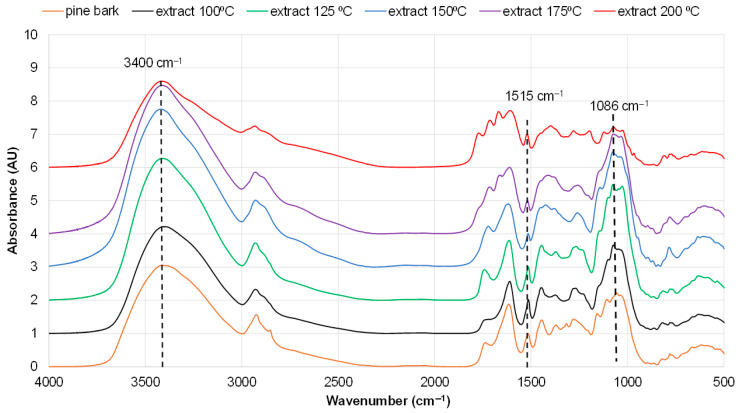
FTIR spectra of initial pine bark and fractions isolated by water extraction at different temperatures.

The FTIR spectrum of the fraction extracted at 150 °C clearly shows the highest absorbance in the regions with peaks at around 1086 cm^−1^ and at about 3400 cm^−1^, generally attributed to carbohydrates. A decrease in absorbance intensity in these regions is observed for fractions extracted at other temperatures. Correspondingly, the lowest absorbance for the fraction isolated at 150 °C was observed at 1515 cm^−1^, attributed to aromatic compounds, indicating the lowest polyphenolic content (Figure 5).

According to FTIR and GC data, the initial bark contains a significantly lower proportion of carbohydrates compared with the extractives obtained from pine bark at 150 °C (Figure 5; Table S2). At the highest yield of 19.6%, the extractives isolated at 150 °C contained 52.4% carbohydrates, recalculated as monomeric sugars (Figure 2). The most abundant monosaccharide was arabinose, followed by glucose and xylose (Table S2).

**Figure 6:**
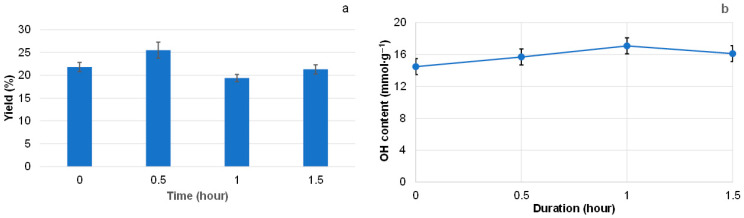
Effect of isothermal heating duration at 150 °C on the yield of pine bark extractives (**a**) in a PARR reactor and on their OH group content (**b**) (at zero duration, the heating was turned off immediately after the reactor reached 150 °C).

Further optimization of the extraction regime was performed at the same extraction temperature of 150 °C, but with a variable duration of isothermal heating (Figure 6).

It was shown that decreasing the isothermal heating duration to 0.5 h increased the yield of extractives to 25% and the carbohydrate content to 57.4%, compared with 19.6% and 52.4%, respectively, for extractives obtained after 1 h of extraction. Clearly, not only the heating temperature but also the duration of heating promotes carbohydrate dehydration. The OH content in extractives isolated at 150 °C was similar across different heating durations, taking into account the standard deviations (Figure 6b). Therefore, isothermal heating at 150 °C for 0.5 h was considered the optimal regime for water extraction of pine bark. The extractives obtained under these conditions are characterized by the high OH group content (~16.0 mmol∙g^−1^), as determined by titration. Together with the highest yield of extractives, this is a positive result, as OH groups play a key role in the modification and liquefaction processes that convert solid extractives into liquid polyols. In these polyols, the terminal OH groups are free from electronic and steric constraints, making them highly reactive toward isocyanates.

### 3.3. Effect of PC/OH Molar Ratio on the Oxypropylation of Pine Bark Extractives and Properties of Ensuing Polyols

In this study, extractives isolated by isothermal heating of pine bark in water at 150 °C for 30 min were transformed into liquid polyols through condensation with PC in the presence of a tertiary amine catalyst. To obtain sufficient material for this process, about 550 g of extractives were prepared, requiring approximately 20 extraction experiments. All of the isolated extractives were combined into a single batch, dissolved in water, and subsequently lyophilized and characterized (Table 2).

In a previous study using model compounds, the authors demonstrated that a temperature range of 150–170 °C can be considered optimal for “green” oxypropylation of biomass components enriched with phenolic and aliphatic OH groups [20]. Lowering the synthesis temperature prolongs the reaction time and leaves part of the PC unconverted. Conversely, excessively high temperatures can promote thermal condensation of the biomass, yielding bio-polyols with excessively high viscosity.

Thermal degradation analysis indicated that the active degradation step of pine bark extractives begins above 180 °C, which justified carrying out the synthesis in the range of 150–170 °C (Figure S2).

In earlier experiments, the DBU/OH molar ratio in the reaction mixture was 0.1, corresponding to 3–6 wt% DBU in the resulting polyol. As a tertiary amine, DBU exhibits catalytic activity in urethane formation, producing highly reactive polyols suitable for spray PU foam systems. To develop polyols better suited for molded systems—with activity adjustable depending on the intended application—the DBU/OH molar ratio was reduced twofold, to 0.05.

**Figure 7:**
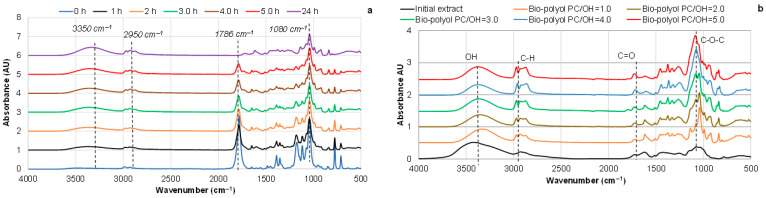
FTIR spectra of bio-polyols obtained by reacting pine bark extractives with PC at 150 °C: (**a**) as a function of reaction duration at a fixed PC/OH ratio of 1.0 and (**b**) as a function of varying PC/OH ratios after complete PC conversion.

The monitoring of FTIR spectra of the reaction products clearly indicates the disappearance of the absorbance band at ~1780 cm^−1^, attributed to the carbonyl group in cyclic carbonate, over the course of the reaction, confirming the complete conversion of PC under the given conditions (Figure 7a and Figure S3). Simultaneously, an increase in absorbance intensity is observed in the 2850–3000 cm^−1^ range, corresponding to symmetric and asymmetric C–H stretching, as well as an increase in the absorbance intensity of OH groups at 3350 cm^−1^ (Figure 7a).

It is known that the PC molecule contains three electrophilic centers: the carbonyl carbon atom and two aliphatic carbon atoms within the cyclic structure, all of which can undergo attack by various nucleophilic agents. In the first case, the PC ring opens via a transcarbonation mechanism, resulting in carbonate structures, while the oxyalkylation mechanism leads to the formation of ether bonds, accompanied by carbon dioxide evolution [21,22,42] (Figure S4).

These mechanisms are responsible for the interaction of PC with aliphatic OH and phenolic groups of pine bark extractive components. However, in the presence of excess PC, this copolymerization process is accompanied by PC self-polymerization, initiated by PC hydrolysis with the formation of propylene glycol, which subsequently participates in further PC ring opening through one of the mechanisms, leading to homopolymer formation [23] (Figure S4).

The presence of a strong absorbance peak at ~1080 cm^−1^ of ether bonds in the reaction products confirms that the oxyalkylation mechanism takes place. The slight decrease in absorbance intensity around 1080 cm^−1^ over time can be attributed to the high initial absorbance of PC at ~1050 cm^−1^, resulting in band overlap during the early stages of the reaction, and its eventual resolution upon complete PC conversion (Figure S3).

**Figure 8:**
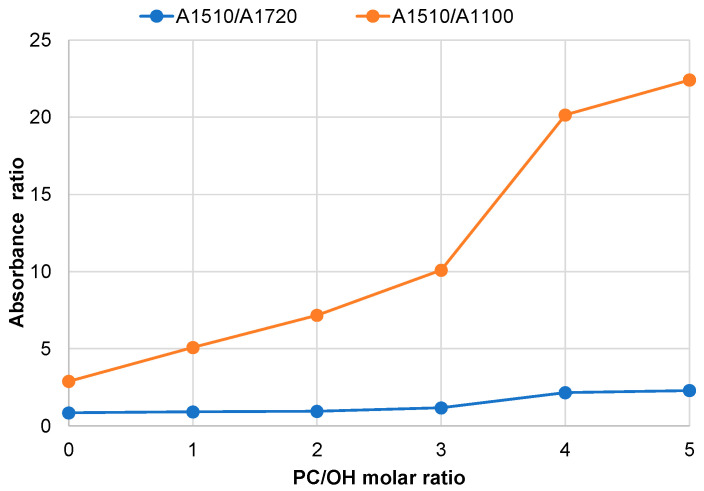
Ratio of the absorbance intensity of aromatics at 1510 cm^−1^ to that of unconjugated carbonyls at 1720 cm^−1^ and ether bonds at 1010 cm^−1^ in the FTIR spectra of bio-polyols as a function of the PC/OH molar ratio.

An increase in the PC/OH ratio steadily enhanced the absorbance peak at ~1080 cm^−1^, accompanied by an increase in the C–H absorbance intensity in the 3000–2850 cm^−1^ region. In addition, the appearance of an absorbance band of unconjugated carbonyl groups at ~1720 cm^−1^ was observed (Figure 7b). This indicates that both oxypropylation and transesterification mechanisms occur during the reaction of pine bark extractives with PC. It is known that aromatic units remain unchanged after oxypropylation [21]. Therefore, the aromatic skeletal vibration at ~1510 cm^−1^ was used as a reference signal to evaluate the contributions of the oxyalkylation and transcarbonation mechanisms in bark extractive processing. The absorbance intensities at ~1080 cm^−1^ and ~1720 cm^−1^, respectively, were normalized to the absorbance intensity at 1510 cm^−1^ (Figure 8).

A drastic increase in the A_1510_/A_1100_ ratio was observed with increasing PC excess. As the PC/OH ratio increased up to 3, the relative content of unconjugated carbonyls in bio-polyols and in the initial extractives remained nearly unchanged. A further increase in PC excess led to only a slight increment in the C=O content of the resulting polyols. This clearly indicates that, regardless of the PC/OH ratio, the modification of pine bark extractives by PC proceeds through both copolymerization and homopolymerization, predominantly via the oxyalkylation mechanism, with only a minor contribution from the transcarbonation reaction.

These findings are consistent with the authors’ earlier results obtained using oregonin-enriched black alder bark extractives [20]. A negligible amount of carbonate units relative to oxypropyl units in the products of kraft lignin—containing both aliphatic and phenolic groups—reacted with PC has also been previously reported [21]. Therefore, all subsequent calculations concerning the composition of biopolyols and their OH content were performed under the assumption of CO_2_ elimination, which accompanies the etherification mechanism in both copolymerization and homopolymerization reactions.

In all experiments, the reaction was carried out at 150 °C for 24 h with FTIR spectra monitoring, as mentioned earlier. If complete PC conversion was not achieved, the synthesis was prolonged with a temperature increase up to 170 °C. It was shown that with increasing PC/OH ratio, the time required for complete PC conversion also increased, due to the decreased concentration of catalyst in the reaction mixture. Additional heating at 170 °C was necessary for all compositions with a PC/OH ratio higher than 2 (Table 3). An increase in both temperature and DBU concentration accelerated PC conversion (Table 3, entries 4; 7; 8).

**Figure 9:**
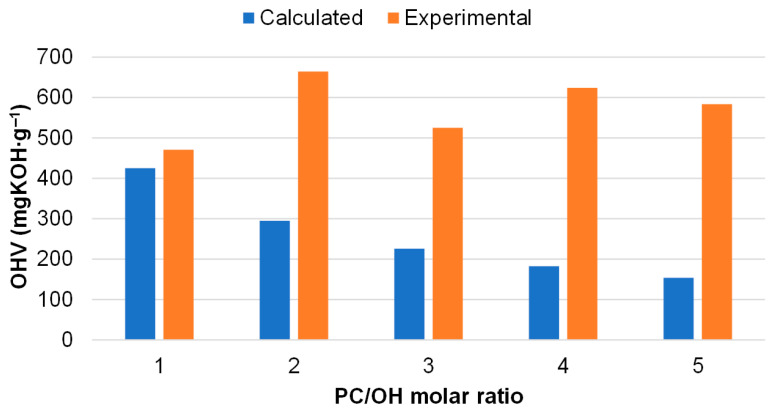
The calculated and experimental OHV of bio-polyols synthesized and different PC/OH molar ratio.

Along with viscosity, the content of OH groups is the dominant characteristic of polyols developed for PU formation. It was shown that the OHV values of bio-polyols were considerably lower compared with those of the extractives (Table 3). This can be explained by the grafting of oxypropyl units (OPU) onto the OH groups of extractive substituents, including carbohydrates and polyphenolics [22]. The lowest OHV, almost twofold lower than in the initial extractives, was observed in the bio-polyol synthesized under stoichiometric conditions. Calculation of the OHV in bio-polyols, assuming that oxypropylation proceeds only via copolymerization and excludes homopolymer formation, showed that, at PC/OH = 1.0, the calculated and experimental OHV values were in close agreement. However, with increasing PC/OH ratio, the deviation between the experimental and calculated results increased significantly (Figure 9).

This can be explained, clearly, by the fact that under stoichiometric conditions, copolymerization is the dominant process, whereas with an excess of PC, homopolymerization of PC occurs with the formation of propanediol and its oligomers, resulting in an increase in the OHV of bio-polyols. For example, the OHV of propanediol and its dimer are 1476 and 837 mg KOH∙g^−1^, respectively, which contributes significantly to the increase in the OHV of bio-polyols. The copolymerization of PC with the components of pine bark extracts is responsible for the increased reactivity with NCO, due to the elimination of steric and electronic constraints of OH groups, despite the overall decrease in their number. In contrast, homopolymerization partly compensates for this decrease by generating OH-enriched propane diols that are highly reactive with isocyanate and act as active co-reagents in extract-based polyols, while simultaneously reducing the polyol viscosity. No acidic groups were detected in the bio-polyols, indicating their conversion into aliphatic hydroxyl groups as a result of the copolymerization reaction.

Those results confirm earlier findings on the oxypropylation of oregonin-enriched black alder bark extractives, showing that the extractives react with a stoichiometric amount of PC independently of the PC/OH ratio. The remaining PC undergoes homopolymerization, forming propane diols and their oligomers, which regulate the viscosity and OH content of the polyols [20].

Viscosity is another key characteristic of polyols that influences the processing parameters of polyurethane foam systems.

All synthesized bio-polyols exhibited non-Newtonian pseudoplastic behavior, with viscosity decreasing as the shear rate increased (Figure S5).

It has been demonstrated that three main factors—PC/OH ratio, processing temperature, and catalyst concentration—affect the viscosity of the bio-polyols (Table 3). Increasing the PC/OH ratio caused the viscosity of the polyols to decrease significantly, due to the reduced weight fraction of copolymers and the increased content of homopolymers. A drastic increase in viscosity was observed when the synthesis temperature was raised from 150 °C to 170 °C, which can be explained by the condensation of a small amount of carbonate anions with terminal OH groups in the copolymer [43].

In contrast, increasing the DBU content in the reaction mixture, at fixed conditions of 150 °C and a PC/OH ratio of 3.0, reduced the viscosity of the polyols by about 1.5-fold (Table 3). These changes were accompanied by an increase in the OHV of the polyols, which can be attributed to the reduced molecular weight of the propane diol oligomers.

Compared with polyols based on black alder bark extractives, oxypropylated pine bark extractives exhibited significantly lower viscosity, indicating that even at the same PC/OH ratio, deep chemical modification does not eliminate the compositional differences between extractives. As usual, polyol polyethers available for PUR foam production have viscosities ranging from 0.3 to 30 Pa∙s and hydroxyl values (OHVs) of 300–800 mg KOH∙g^−1^, depending on the functionality and molecular weight of the hydroxyl-containing starters [44]. Therefore, in terms of the viscosity of the polyols derived from pine bark extractives, the most suitable conditions are a PC/OH molar ratio of 3, a main synthesis temperature of 150 °C, and a DBU/OH molar ratio of 0.05.

Reducing the PC excess leads to polyols with a high biomass content, though it also results in excessively high viscosity. Conversely, increasing the PC/OH ratio above 3 decreases both the viscosity and the biomass content of the resulting bio-polyols. Increasing the DBU/OH ratio to 0.1 raises the weight fraction of tertiary amine in the polyols from 3.1% to 6% and the OHV from 527 to 710 mg KOH∙g^−1^. While this produces highly reactive polyols, it also creates challenges in controlling their reactivity in molded PUR foam systems.

### 3.4. Characteristics of PUR Foams on the Basis of Synthesized Bio-Polyols

#### 3.4.1. Effect of the Substitution Extent of Commercial Polyols with Bio-Polyol on the Foaming Behavior, Morphology, Mechanical Properties, and Thermal Degradation of PUR Foams

The properties of PUR foams containing pine bark extractives-based polyols were compared with those of reference PUR foams based on the commercial polyethers Lupranol 3300, Lupranol 3422, and their 1:1 mixture (Table 4). In the initial stage of the study, 50% of Lupranol 3300 by weight was replaced with bio-polyols synthesized under different conditions (Nr. 3–6 in Table 3). In all recipes, the NCO/OH molar ratio was kept constant at 1.15.

Except for the reference compositions (R1–R3), which are based exclusively on commercial polyols, the weight fraction of bio-polyols in the polyol component of the other formulations was 50% (Table 3).

**Figure 10:**
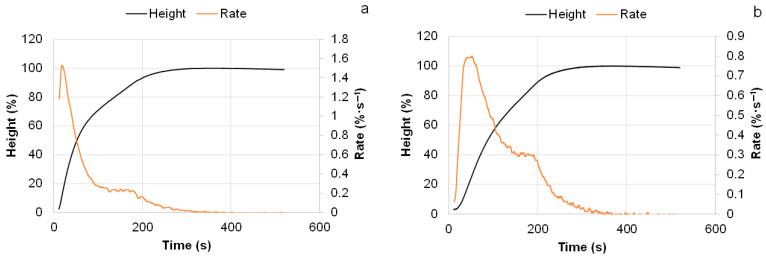
The height and rise rate vs. foaming time for R3 (**a**) and BP3 (**b**) PUR foam compositions (sample abbreviations are consistent with Table 4).

The dynamics of the foaming process were studied using the Universal Foam Qualification System FOAMAT 285, which automatically records foam height during foaming (Figure 10). A 50% substitution of commercial polyol with bio-polyols synthesized at PC/OH = 2–3 did not significantly affect the activity of the PUR foam systems.

For example, the start time and the time required to reach 50% of the final foam height for the R3 system, based on commercial polyol blends, were 18 and 54 s, respectively. For the BP2 and BP3 compositions, these parameters were in the ranges of 18–21 s and 52–59 s, respectively. In contrast, the use of bio-polyols synthesized at PC/OH = 4–5 led to variations in the start time and 50% growth time within the ranges of 25–31 s and 115–148 s, respectively, indicating reduced activity of the PU systems. This decrease can be attributed to the lower concentration of DBU in the bio-polyols with increasing PC/OH molar ratio during their synthesis (Table 3).

The substitution of commercial polyols by bio-polyols does not negatively affect the morphological structure of foams. The volumetric content of the closed cell in reference foams varied in the range of 88.3–87.7% vs. 88.1–91.9% in bio-polyols containing samples (Figure S6).

The apparent densities of the PUR foams prepared according to the formulations presented in Table 4 ranged from 45 to 55 kg∙m^−3^. To eliminate the influence of density on the physical–mechanical properties when comparing different materials, the compressive strength and Young’s modulus of all PUR foams were normalized to a reference density of 50 kg∙m^−3^, following the regressions described by Hawkins [45].

**Figure 11:**
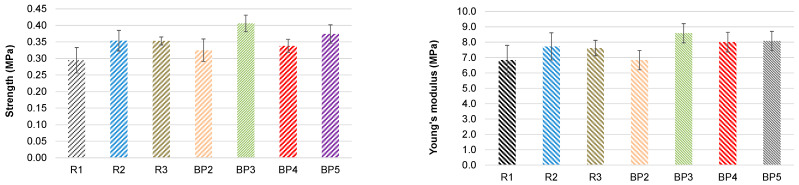
Normalized at apparent density of 50 kg∙m^−3^ compression characteristics in foaming direction of PUR foams, references and with polyol systems substituted by 50% on bio-polyol (sample abbreviations are consistent with Table 4).

The incorporation of the bio-polyol (PC/OH = 2), which is the most enriched with biomass, into the PU composition led to a decrease in both the compressive strength and Young’s modulus of the materials compared with foams based on the pure six-functional polyol Lupranol 3422 and the mixture of the two commercial polyols (Figure 11). This effect is likely attributed to the high viscosity of the bio-polyol, which hinders uniform mixing of the components and may result in the formation of defects in the chemical and morphological structure of the materials.

The bio-polyol synthesized at 150 °C with PC/OH = 3 and DBU/OH = 0.05 showed a clear trend of improvement in compressive properties, confirming these parameters as optimal for pine bark extractive-based bio-polyol synthesis. By contrast, bio-polyols obtained at PC/OH ratios of 4 and 5 had only negligible effects on the compression characteristics of PUR foams (Figure 11).

In the next series of experiments, commercial polyols were completely substituted with bio-polyols synthesized at the optimal PC/OH ratio of 3, but differing in synthesis temperature and catalyst content. No commercial urethane formation catalyst was additionally introduced in bio-polyol based system (Table 5).

**Figure 12:**
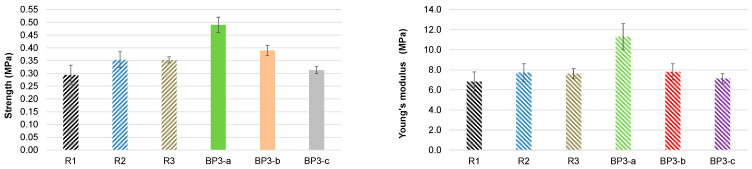
Compressive characteristics in the foaming direction of PUR foams normalized to an apparent density of 50 kg∙m^−3^ for reference and 100% bio-polyol systems (sample abbreviations are consistent with Table 4).

Nevertheless, despite the presence of the DBU catalyst in the bio-polyols, the activity of the bio-polyol-based system was lower compared with the reference systems containing the commercial catalytic system Polycat. The start times for the bio-polyol-based PUR foam systems were 95 and 59 s when the DBU content in the bio-polyol was 3.1% and 6.0%, respectively (Table 3). Therefore, the bio-polyol-based compositions can be considered suitable for mold PU systems, with the possibility of tuning their activity by adding a commercial catalytic system. Similarly to the first experimental series, the BP3-a PUR foam composition based on the bio-polyol synthesized at 150 °C with PC/OH = 3 and DBU/OH = 0.05 exhibited the best compressive characteristics, exceeding those of the commercial polyol-based materials by about 30–35% (Figure 12).

At a fixed PC/OH ratio of 3, increasing the DBU content in the reaction mixture and the synthesis temperature led to a decrease in the compressive properties of the resulting PUR foams. The increase in synthesis temperature had a more pronounced negative effect. This is likely due to the high viscosity of bio-polyol Nr. 8, which hinders proper mixing of the components and promotes the formation of structural defects. A significant increase in OHV, accompanied by a corresponding decrease in the viscosity of bio-polyol Nr. 7, compared with bio-polyol Nr. 4, was observed (Table 4). These results suggest that a twofold increase in the DBU/OH molar ratio to 0.1 further promotes the formation of low-molecular-weight bifunctional propane diols, reducing the average functionality of the bio-polyols and, consequently, the compressive characteristics of the BP3-b composition compared with the BP3-a sample. It was established that bio-polyols derived from black alder bark and pine bark extractives, both synthesized under optimal conditions, produce PU foams with similar compression characteristics, significantly exceeding those of PU foams based on commercial polyols [20,32].

**Figure 13:**
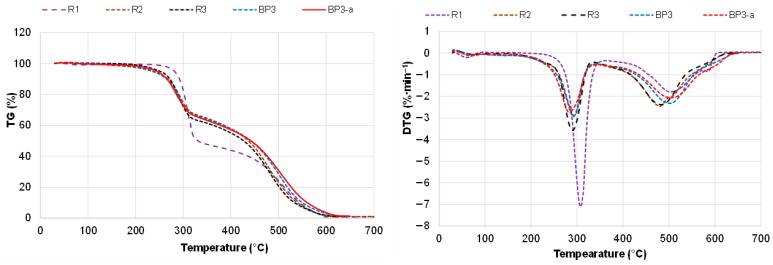
TG and DTG curves in air for references and bio-polyol containing PUR foams at 50% (BP3) and 100% (BP3-a) substitution of commercial polyols (sample abbreviations are consistent with Table 4 and Table 5).

The effect of substituting commercial polyols with bio-polyols derived from pine bark extractives, synthesized under optimal conditions, on the thermo-oxidative stability of the resulting PUR foams was studied using non-isothermal thermogravimetric analysis in air, in comparison with foams based on commercial polyols (Figure 13).

As shown, the incorporation of bio-polyol into the PUR composition led to a decrease in both the peak degradation rate and the fraction of material degraded in the volatile formation zone (200–400 °C), while simultaneously increasing the fraction decomposed in the 400–650 °C range, where char oxidation and combustion predominate. This effect was most pronounced in comparison with the material based on the trifunctional Lupranol 3300. However, compared with materials containing the hexafunctional polyol Lupranol 3422, the bio-polyol-based material also exhibited a lower peak of degradation rate, a higher temperature at 50% weight loss, and a 1.5-fold higher char residue yield at 500 °C (Figure 13; Table 6). These results indicate a more pronounced char-forming tendency during the thermal-oxidative degradation of the bio-polyol-based foam compared with the reference material. Considering the well-known protective role of the char layer during combustion, a reduction in the flammability of bio-polyol-based foams relative to those based on commercial polyols can be suggested [46].

#### 3.4.2. The Extracted Pine Bark as a Natural Filler of Bio-Polyol Based PUR Foams

Alongside the utilization of bio-polyols, the incorporation of lignocellulosic fillers into the PUR foam matrix as substitutes for petroleum-based compounds is a highly relevant approach due to their wide availability, low cost, and positive effects on several foam properties [27,47,48].

**Figure 14:**
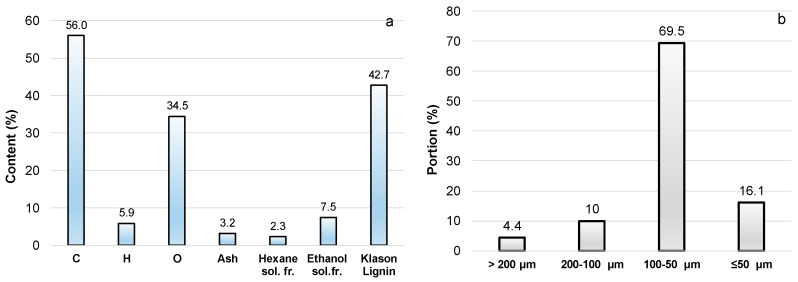
Composition on DM (**a**) and particle size distribution (**b**) of ground extracted bark.

In this study, pine bark remaining at approximately 75% by weight after the isolation of carbohydrate-enriched extractives was investigated as a natural filler for bio-polyol-based PU compositions. The extracted bark contains 42.7% KL versus 32.3% in the initial pine bark, has lower contents of hexane- and ethanol-soluble fractions, and higher carbon content (Table 1; Figure 14). The char residue yield from the pyrolysis of the extracted bark was approximately 30% (Figure S7). Therefore, it can be proposed that the extracted bark may act as a promoter of char formation when incorporated into a PU matrix under thermal stress and will not increase the flammability of the material.

Ground and oven-dried extracted bark was incorporated into the BP3-a composition in amounts of 1–40 pbw per 100 pbw of bio-polyol. Increasing the filler content above 40 pbw resulted in excessively high viscosity of the polyol system, which hindered proper mixing of the ingredients. Consequently, the filler content in the PU matrix ranged from 1.8% to 13.0%, while the total biomass content, including the extractives in the bio-polyol, ranged from 11.7% to 21.8% (Figure S8).

As shown in previous studies, the incorporation of black alder bark biomass can retard the foaming process [32]. Considering that the bio-polyol-based PUR system does not contain an additional catalyst, a small amount of Catalyst Polycat (0.15 pbw) was added to the pine bark filled compositions.

**Figure 15:**
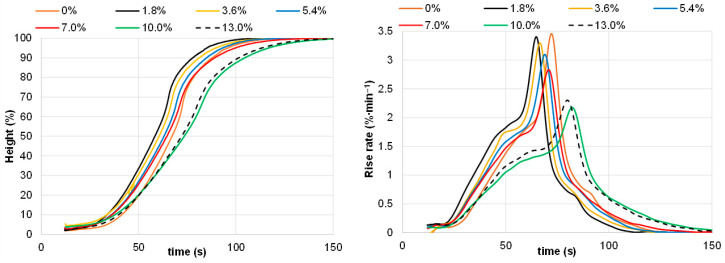
Effect of extracted pine bark content in the PU matrix on the dependence of foam height and rise rate on foaming time for the bio-polyol-based PUR foam system.

It was shown that in the filler content range of 1.8–7%, the pine bark filler did not retard the foaming process, in contrast to a more expressed retardation effect observed earlier for the black alder bark-derived filler [32]. Moreover, within this range of pine bark filler content, a slight trend toward increased activity of the filled PUR foam systems compared with the initial non-filled composition was noticeable (Figure 15). Clearly, this can be explained by the fact that OH groups on the surface of lignocellulosic matrix are prone to bind with free isocyanate groups, therefore increasing compatibility with the PU matrix [47]. According to tests performed using the Universal Foam Qualification System FOAMAT 285, the start time, 50% height rise time, and full rise time for systems containing 0–7% of filler was 29 ± 2, 62 ± 4, and 100 ± 6 s, respectively. Increasing the filler content in the PU matrix to 10–13% led to a more noticeable increase in the 50% height rise and full rise times, which reached 75 and 130 s, respectively.

Similarly, no effect on foaming kinetics was observed in PUR foam formulations filled with up to 10% wood sawdust [47]. It was established that the incorporation of pine bark filler did not negatively affect the morphological structure of PUR foams in terms of volumetric closed-cell content. The closed-cell content in the filled samples ranged from 90.3 to 91.5 vol.% compared with 88.8 vol.% in the bio-polyol-based unfilled foam.

A negligible increase in the apparent density of PUR foams—from 40.5 to 43.5 kg·m^−3^—was observed upon incorporation of the bark filler into the formulation (Figure S9). These results are consistent with the absence of a significant retardation effect of the filler on the foaming process, which prevents a substantial decrease in the temperature inside the rising block and, consequently, limits the increase in apparent density.

**Figure 16:**
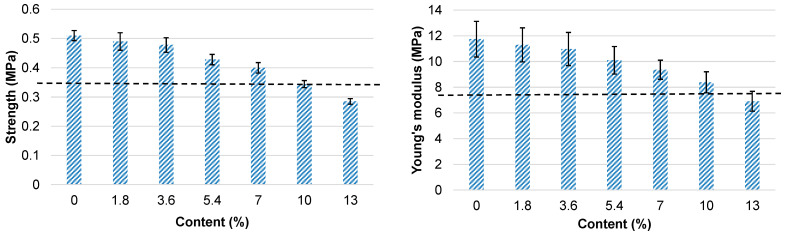
Normalized compressive characteristics of bio-polyol-based PUR foams in the foaming direction as a function of natural filler content (the corresponding values for the unfilled commercial polyol-based PUR foam R3 are indicated by the dashed line).

In contrast, the incorporation of the filler resulted in a continuous decrease in both compressive strength and modulus (Figure S10). At the maximum filler content of 13%, both the compressive strength and modulus of the material were about 65% of those of the bio-polyol-based unfilled foam. To enable comparison of the mechanical characteristics of the bio-polyol-based filled foams with those of the commercial polyol-based material, the mechanical properties of the filled foams were also normalized to a reference density of 50 kg·m^−3^ (Figure 16).

Despite the negative effect of extracted bark as a filler on the compressive properties of the filled materials, it was shown that, with up to a 7% filler content in the PU matrix, the compressive properties of the bio-based PUR foams are higher, while at 10% filler content they are comparable to those of the commercial polyol-based PUR foam (Figure 16).

The high thermal insulation ability of rigid PUR foams is one of the main reasons for the wide practical application of this material. The thermal conductivity coefficient (λ) for the reference (R3), the bio-polyol-based foam, and the bio-polyol-based PUR foam containing 10% filler (relative to the PU matrix) was determined to be 0.0222, 0.0207, and 0.0253 W·m^−1^·K^−1^, respectively, at apparent densities of 52, 48, and 50 kg·m^−3^. The bio-polyol-based foam exhibited slightly improved thermal insulation performance compared with the reference material. This improvement can be attributed to its smaller average cell size and/or a more uniform cell size distribution, which reduces the radiative contribution to the total heat transfer within the bio-polyol-based foam [49].

This effect is more pronounced in foams with a higher volumetric fraction of the gaseous phase, i.e., in lower-density foams. A more detailed investigation of bio-polyol-based foam morphology is under planning. The incorporation of 10% pine bark filler (based on the PU matrix) increased the λ value of the filled material by about 19% compared with the unfilled foam, despite both materials having similar closed-cell contents (~91 vol.%). Similar results have been reported for PUR foams filled with glass powder, rice straw fiber, walnut shell, and sawdust powder, where the increased thermal conductivity was attributed to enhanced heat transport through the solid phase [50,51].

The obtained results are satisfactory, considering that the thermal conductivity coefficients of the bio-polyol-based foams, including those with filler, meet the requirements for commercial PUR foams, which typically range between 0.020 and 0.030 W∙m^−1^∙K^−1^ [52,53].

#### 3.4.3. Cone Calorimetric Tests of Reference and Pine Bark Biomass-Containing PUR Foams

Cone calorimeter tests were performed to evaluate the effect of pine bark-derived bio-polyol and filler incorporated into the PU matrix on the combustion behavior of biomass-containing PUR foams. This test is recognized as one of the most comprehensive modern bench-scale methods for simulating real fire conditions [54,55]. A cone calorimeter is used for fire testing of a wide range of modern materials, such as silicone rubber foams filled with carbon nanotubes [56]. The bio-polyol-based PUR foam BP3-a (Table 3), with 100% substitution of commercial polyol, both unfilled and containing 10% of extracted bark as a filler, was tested against the PUR foam system R3 (Table 4), developed on the basis of commercial polyols.

**Figure 17:**
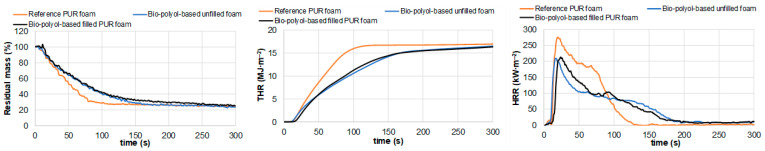
Residual mass, total heat release (THR), and heat release rate (HRR) as a function of combustion time for reference and bio-polyol-based PUR foams, both unfilled and filled compositions.

Key combustion characteristics of the materials—including time to ignition (TTI), time to flameout (TFO), average mass loss rate (Av-MLR), total heat release (THR), peak heat release rate (PHRR), maximum average rate of heat emission (MARHE), average effective heat of combustion (Av-EHC), total smoke release (TSR), smoke release rate (SRR), and average yields of CO_2_ (Av-CO_2_Y) and CO (Av-COY)—were evaluated (Table 7, Figure 16 and Figure 17) [57].

The combustion behaviors of unfilled and filled bio-polyol-based PUR foams were found to be similar to each other but differed significantly from that of the reference PUR foam. It was found that the mass losses during the combustion tests for all samples under study varied in the range of 80.4–84.2%. These losses were accompanied by THR values ranging from 17.4 to 18.8 MJ·m^−2^. Among the tested samples, a slight increase in both Δm and THR values—up to 84.2% and 18.8 MJ·m^−2^, respectively—was observed for the bio-polyol-based PUR foam containing 10% filler.

However, the flameout time (TFO) for both bio-polyol-based PUR foams was 1.25–1.35 times longer compared with that of the reference foam. This extended TFO was accompanied by an approximately 1.5-fold lower average mass loss rate (Av-MLR) relative to the reference material, resulting in significantly lower heat emission. For example, due to these different combustion rates, 90% of the maximum THR for the bio-polyol-based PUR foams was emitted after approximately 250 s of testing, compared with only 100 s for the reference sample.

The heat release rate is one of the most important characteristics for fire hazard evaluation, as it governs the rate of fire growth, including heat release and production of gaseous species [58]. It was established that the peak heat release rate (PHRR) and maximum average rate of heat emission (MARHE) for bio-polyol-based PUR foams were lower than those of the reference material by approximately 25% and 30%, respectively. The HRR, THR, and MARHE data, supported by the weight-loss dynamics of the tested samples, indicate slower degradation of the bio-polyol-based PUR foams compared with the reference foam. This suggests lower flammability, a reduced tendency for fire development, and a decreased potential for fire hazard relative to the reference material (Table 7; Figure 17) [57,59].

**Figure 18:**
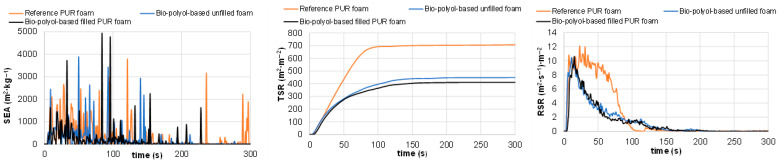
Smoke emission area (SEA), total smoke release (TSR), and smoke release rate (SRR) values as a function of combustion time for reference and bio-polyol-based PUR foams, both unfilled and filled compositions.

It was observed that the higher degradation rate of the reference material was accompanied by a significantly greater content of non-flammable by-products, such as smoke, in the combustion products of the reference PUR foam compared with the bio-polyol-based foam (Table 7; Figure 18). The total smoke released during the combustion of the reference PUR foam was approximately 38% higher than that of the bio-polyol-based foam.

This indicates that a larger portion of carbon in the reference foam was converted into particulate matter emitted with the gaseous products of PU thermal degradation. Unlike combustible volatiles, this particulate matter was not oxidized to release heat but remained as a solid air pollutant. As a result, the effective heat of combustion and CO_2_ and CO emissions for the reference sample were lower compared with the corresponding values for the bio-polyol-based PUR foam (Table 7). However, the contribution to air pollution from solid products of incomplete combustion was considerably higher.

Finally, it should be noted that the substitution of commercial polyols with pine bark extractive-derived polyols was the primary factor responsible for the decreased flammability of PUR foams. An increase in biomass content due to the incorporation of extracted bark as a filler did not provide an additional reduction in the flammability of the material.

## 4. Conclusions

In this study, the potential of pine bark, a multi-ton forestry by-product, was comprehensively evaluated as a renewable and sustainable resource for PUR foam production. It was established that pressurized water extraction under optimal conditions, including isothermal heating at 150 °C for 30 min, allows for the production of extractives with maximal yield, carbohydrate and hydroxyl group contents, reaching 25%, 57%, and 16.0 mmol∙g^−1^, respectively. Deviations from the optimal extraction temperature, either lower or higher, result in decreased yield and hydroxyl group content in the extractives. The isolated pine bark extractives, enriched in hydroxyl groups, were subsequently used as precursors for bio-polyol synthesis, employing propylene carbonate as a “green” oxypropylation agent. A PC/OH molar ratio of 3 and a synthesis temperature of 150 °C were found to be optimal for bio-polyol production. Under these conditions, a bio-polyol with an OHV of 527 mg KOH·g^−1^, a viscosity of 14.9 mPa·s at 25 °C, and a biomass content of approximately 27% was synthesized.

Substituting commercial polyols with this bio-polyol allowed an increase of up to 35% in the mechanical properties of the foam at compression along the foaming direction at the same apparent density, while simultaneously improving its thermal insulation characteristics by 9% compared with reference materials based on commercial polyether polyols Lupranol 3300 and Lupranol 3422. Moreover, the bio-polyol-based PUR foam demonstrated better thermal stability in air, exhibiting a reduced maximum degradation rate and a 1.5-fold higher char residue at 500 °C.

The introduction of extracted bark as a filler in amounts of 1.8–13% in a bio-polyol-based PU matrix does not significantly affect the apparent density or closed-cell content, increasing the biomass content in the material to approximately 22%. Nevertheless, a decrease in compression properties of up to 35% and an increase in the thermal isolation coefficient from 0.0207 to 0.0253 W·m^−1^∙K^−1^ were observed when compared with the unfilled material. However, the filled compositions exhibited improved compression characteristics at filler contents of up to 10% compared with reference PUR foams.

Cone calorimeter tests showed that the bio-polyol-based PUR foam exhibited lower values of peak heat release rate (PHRR), maximum average rate of heat emission (MARHE), and total smoke release (TSR) by 25%, 30%, and 38%, respectively, compared with the reference material. These improvements were not further enhanced in the filled compositions. Combustion tests indicates that both unfilled and filled bio-polyol-based PUR foams have lower flammability and a reduced tendency for fire development compared with the reference material.

Overall, the results clearly indicate that pine bark is a promising natural resource for complex applications in the production of bio-based PUR foams, yielding materials whose main characteristics are comparable to or exceed those of foams based on commercial fossil-derived polyols.

## Figures and Tables

**Table 1:** The elemental composition, ash, Klason lignin and extractives content in initial pine bark.

Content on DM, %	Mean ^1^
Carbon	52.8 ± 0.5
Hydrogen	6.1 ± 0.05
Nitrogen	0.42 ± 0.04
Ash	2.6 ± 0.2
Lignin (by TAPPI 222 2-om):	
Acid insoluble (Klason Lignin)	31.7 ± 0.3
Acid soluble	1.5 ± 0.1
Total	33.2 ± 0.3
Extractives ^2^:	
Hexane soluble	3.2 ± 0.2
Ethanol (96%) soluble	16.8 ± 0.3
Total	21.0 ± 0.4
Monomeric carbohydrates content ^3^	38.5 ± 2.4

^1^ Including Std of three repeated measurements. ^2^ Soxhlet extraction for 8 h. ^3^ After complete hydrolysis (Table S2).

**Table 2:** Characteristics of pine bark extractives used as a precursor for bio-polyol synthesis.

Content on DM	Mean ^2^
Carbon (%)	41.2 ± 0.5
Hydrogen (%)	6.10 ± 0.05
Nitrogen (%)	0.42 ± 0.02
Ash (%)	2.6 ± 0.2
TPC (GAE g∙g^−1^)	0.08 ± 0.02
Monomeric carbohydrate (%) ^1^	57.4 ± 2.2
ΣOH group (mmol∙g^−1^)	16.0 ± 0.1

^1^ After complete hydrolysis; ^2^ including Std of three repeated measurements.

**Table 3:** The effect of PC/OH molar ratio on composition and properties of ensuing bio-polyols.

Nr. ^1^	PC/OH Molar Ratio	PC/Extract Weight Ratio	Content in Bio-Polyol (% *w/w*) ^2^		Reaction Time (h)		OHV (mg KOH∙g^−1^)	Viscosity (25 °C) at 50 s^−1^ (Pa∙s)	H_2_O by K.F (%)
			Biomass	DBU	150 °C	170 °C			
1	-	-	100.0	-	-	-	846 ± 21	-	-
2	1.0	1.5	50.2	5.8	24	0	471 ± 19	>1000	0.25
3	2.0	3.1	34.9	4.0	24	6	664 ± 21	85.1 ± 5.1	0.20
4	3.0	4.6	26.7	3.1	24	8	527 ± 18	14.9 ± 2.1	0.16
5	4.0	6.2	21.6	2.5	24	16	624 ± 27	9.9 ± 1.6	0.13
6	5.0	7.7	18.2	2.1	24	24	584 ± 39	8.6 ± 0.6	0.08
7 ^3^	3.0	4.6	26.7	6.0	24	0	710 ± 17	9.2 ± 1	0.09
8 ^4^	3.0	4.6	26.7	3.1	0	24	592 ± 21	210 ± 250	0.11

Note: ^1^ Nr. 1-initial extract, Nr. 2–8-bio-polyols; ^2^ calculation assuming only etherification mechanism for PC cycle opening; ^3^ DBU/OH molar ratio equal 0.1 instead of that 0.05 for other samples; ^4^ reaction performed at 170 °C only.

**Table 4:** Composition of references and bio-polyol containing PUR foams.

Position	R1	R2	50% Substitution of Lupranol 3300				
			R3	BP2	BP3	BP4	BP5
Composition (pbw):							
Lupranol 3300	100	-	50	50	50	50	50
Lupranol 3422	-	100	50	-	-	-	-
Bio-polyol (PC/OH = 2)	-	-	-	50	-	-	-
Bio-polyol (PC/OH = 3)	-	-	-	-	50	-	-
Bio-polyol (PC/OH = 4)	-	-	-	-	-	50	-
Bio-polyol (PC/OH = 5)	-	-	-	-	-	-	50
Water	0.50	0.50	0.50	0.50	0.50	0.50	0.50
Catalyst Polycat	0.7	0.7	0.7	0.7	0.7	0.7	0.7
Surfactant, Niax Silicone	1.5	1.5	1.5	1.5	1.5	1.5	1.5
Blowing agent, Opteon ^TM^ 1100	20	20	20	20	20	20	20
Plasticizer TCPP	11	11	11	11	11	11	11
Isocyanate pMDI	119.5	144.1	133.7	155.6	136.9	150.1	144.7
B/A ratio ^1^	0.89	1.08	1.0	1.16	1.02	1.12	1.08

Notes: ^1^ All formulation ingredients, except pMDI, were defined as component A, while pMDI was defined as a component B.

**Table 5:** Composition of PUR foams with complete (100%) substitution of commercial polyols with bio-polyols.

Position	PUR Foam System		
	BP3-a	BP3-b	BP3-c
Composition (pbw):			
^1^ Bio-polyol Nr. 4	100	-	-
^1^ Bio-polyol Nr. 7	-	100	-
^1^ Bio-polyol Nr. 8	-	-	100
Water	0.50	0.50	0.50
Catalyst Polycat	-	-	-
Surfactant, Niax Silicone	1.5	1.5	1.5
Blowing agent, Opteon ^TM^ 1100	20	20	20
Plasticizer TCPP	11	11	11
Isocyanate pMDI	154.3	201.4	172.2
B/A ratio	1.16	1.54	1.29

Note: ^1^ Sample abbreviations are consistent with Table 3.

**Table 6:** Parameters of thermal oxidative degradation of reference and bio-polyol-based PUR foams according to non-isothermal TG/DTG.

Sample	T_5%_ (°C) ^1^	$\frac{dm}{dt}$_max_ (%∙min^−1^) ^2^	T_max_ (°C) ^3^	T_50%_ (°C) ^4^	Δm_500°C_ (%) ^5^
R1	282 ± 6	7.1 ± 0.4	308 ± 8	350 ± 10	24.2 ± 1.0
R2	233 ± 5	2.9 ± 0.2	294 ± 4	427 ± 6	20.8 ± 0.8
R3	251 ± 5	3.6 ± 0.3	291 ± 5	425 ± 10	20.7 ± 0.7
BP3	236 ± 2	2.9 ± 0.2	291 ± 4	440 ± 8	28.5 ± 1.2
BP3a	244 ± 5	2.5 ± 0.1	284 ± 6	442 ± 7	30.8 ± 0.8

Note: ^1^ temperature of 5% weight loss; ^2^ maximal degradation rate; ^3^ temperature of maximal degradation rate; ^4^ temperature of 50% weight loss; ^5^ residual weights at 500 °C.

**Table 7:** The results of cone calorimetric tests of PUR foams.

Parameters	Abbreviation	PUR Foam Compositions		
		Reference	Bio-Polyol-Based Unfilled	Bio-Polyol-Based Filled
Apparent density, kg∙m^−3^	-	52 ± 1	48 ± 1	50 ± 1
Time to ignition, s	TTI	4.3 ± 0.5	3.3 ± 0.5	5.7 ± 1
Time to flameout, s	TFO	133 ± 10	166 ± 14	180 ± 14
Mass loss, %	Δm	81.0 ± 1.3	80.4 ± 0.8	84.2 ± 3.2
Average mass loss rate, %∙s^−1^	Av-MLR	0.66 ± 0.04	0.44 ± 0.04	0.43 ± 0.05
Total heat release, MJ∙m^−2^	THR	17.4 ± 0.6	17.8 ± 0.4	18.8 ± 0.8
Peak heat release rate, kW∙m^−2^	PHRR	278 ± 2	208 ± 9	207 ± 5
Maximum average rate of heat emission, kW∙m^−2^	MARHE	175.8 ± 7.6	124.4 ± 5.6	124.7 ± 3.3
Average effective heat of combustion, MJ∙kg^−1^	Av-EHC	15.4 ± 1	18.5 ± 0.4	18.1 ± 0.3
Total smoke release, m^2^∙m^−2^	TSR	764 ± 51	476 ± 21	479 ± 31
Average carbon dioxide yield, kg∙kg^−1^	Av-CO_2_Y	1.70 ± 0.02	2.10 ± 0.05	2.06 ± 0.11
Average carbon monoxide yield, kg∙kg^−1^	Av-COY	0.082 ± 0.002	0.132 ± 0.011	0.145 ± 0.015

## Data Availability

The original contributions presented in this study are included in the article/Supplementary Material. Further inquiries can be directed to the corresponding author.

## Supplementary Materials

The following supporting information can be downloaded at https://www.mdpi.com/article/10.3390/polym18010096/s1, Table S1: UHPLC–MS/MS identification of compounds in pine bark water extract obtained at 150 °C; Table S2: The monomeric carbohydrate content in completely hydrolyzed pine bark and pine bark extractives in dependence of extraction temperature; Figure S1: UHPLC chromatograms of pine bark water extract isolated at 150 °C; Figure S2: The TG/DTG curves in argon atmosphere of pine bark extractives; Figure S3: The FTIR spectra of PC; Figure S4: The pathways of the PC ring opening in its reaction with hydroxyl-containing compounds; Figure S5: Share rate–viscosity curves of bio-polyol synthesized at different PC/OH ratio; Figure S6: Closed-cell content by volume in PUR foams, references and with polyol systems substituted by 50% on bio-polyol (sample abbreviations are consistent with Table 4); Figure S7: TG and DTG curves of extracted bark in argon and air; Figure S8: Content of bio-polyol, natural filler, and total biomass in the PU matrix as a function of extracted bark addition; Figure S9: The effect of extracted bark content in the PU matrix on the apparent density of filled PUR foams and their mechanical characteristics under compression in the foaming direction.

## Abbreviations

The following abbreviations are used in this manuscript:

PU	Polyurethane
PUR foam	Rigid polyurethane foam
PO	Propylene oxide
PC	Propylene carbonate
pMDI	Polymeric methane disicyanate
TCPP	Tris(1-chloro-2-propyl)phosphate
DBU	1,8-diazabicyclo[5.4.0]undec-7-ene
OHV	Hydroxyl value
pbw	Part by weight
KL	Klason lignin
TTI	Time to ignition
TFO	Time to flameout
THR	Total heat release
TSR	Total smoke release
PHRR	Peak heat release rate
MARHE	Maximal average heat emission
Av-EHC	Average effective heat of combustion
Av-CO_2_Y	Average CO_2_ emission
Av-COY	Average CO emission
