# Supplementary material for: Pine Bark as a Lignocellulosic Resource for Polyurethane Production: An Evaluation
Source: Polymers (Basel). 2025 Dec 29;18(1):96. doi: 10.3390/polym18010096 (PMC12787510; doi:10.3390/polym18010096)
Supplement: Supplementary file 1 [file polymers-18-00096-s001.zip › polymers-4041114-supplementary.pdf]

Pine Bark as a Lignocellulosic Resource for Polyurethane Production: An Evaluation

Alexander Arshanitsa*, Matiss Pals, Alexandra Vjalikova, Laima Vevere, Oskars Bikovens, and Lilija Jashina

Latvian State Institute of Wood Chemistry, Dzerbenes Street 27, LV-1006, Riga, arshanica@edi.lv (A.A); matiss.pals@kki.lv (M.P); alexandra vjalikova (A.V); laima.vevere@kki.lv (L.V); oskars.bikovens@kki.lv; lilija_jasina@inbox.lv (L.J).

* Correspondence: arshanica@edi.lv; Tel.:+371 29472942

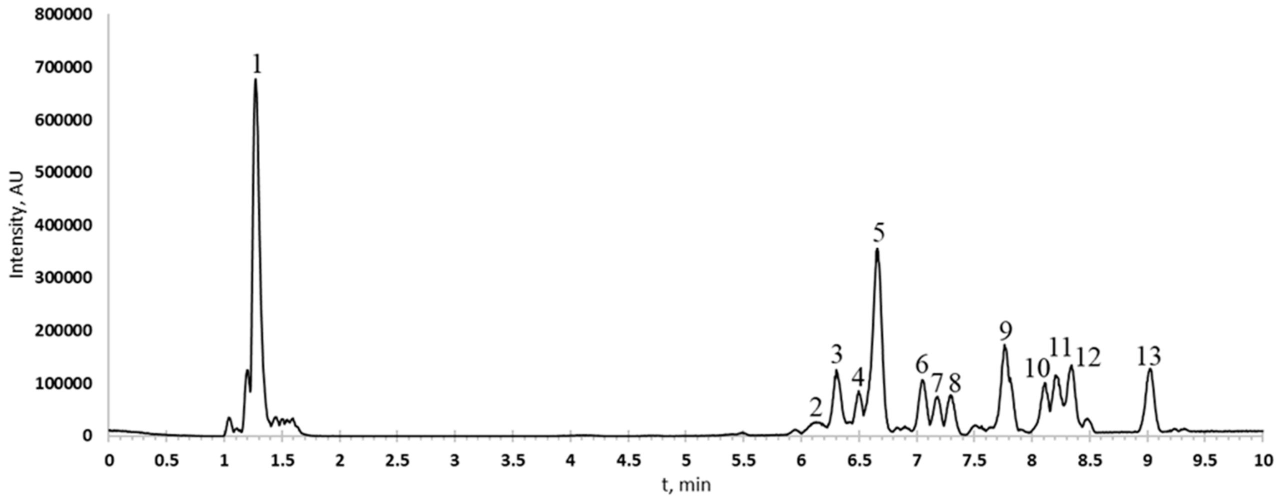

Figure S1. UHPLC chromatograms of pine bark water extract isolated at 150 °C.

Table S1. UHPLC–MS/MS identification of compounds in pine bark water extract obtained at 150 °C.

Nr	[M-H]-	RT(min)	Identification
1	179.15	1.302	Carbohydrates
2	458.084	6.177	Epigallocatechin gallate
3	577.137	6.315	Proanthocyanidin B-dimer
4	315.11	6.517	Pinoquercetin
5	289.057	6.767	Epicatechin
6	301.11	7.055	Quercetin
7	579.152	7.197	Chalcan-flavan-3-ol dimer
8	285.048	7.316	Kaempferol
9	609.158	7.781	Rutin

10	575.12	8.125	A-type procyanidin dimer
11	575.12	8.236	A-type procyanidin dimer
12	575.12	8.358	A-type procyanidin dimer
13	865.198	9.031	Proanthocyanidin B-trimer

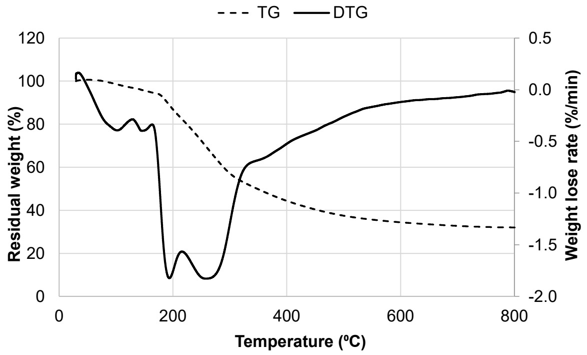

Figure S2. The TG/DTG curves in argon atmosphere of pine bark extractives.

Table S2. The monomeric carbohydrate content in completely hydrolyzed pine bark and pine bark extractives.

T (°C)	Content on DM (%)						
	Glc	Gal	Man	Xyl	Ara	Rha	Σ
Pine bark	22.5±2.3	2.0±0.1	2.6±0.3	3.7±0.1	7.0±0.1	0.7±0.2	38.5±2.4
100	14.0±0.5	1.5±0.1	2.0±0.2	1.0±0.2	12.5±1.0	1.6±0.2	32.3±1.2
125	10.6±0.9	2.2±0.2	1.8±0.2	1.8±0.1	23.2±1.3	1.5±0.1	41.1±1.6
150	12.1±1.1	4.4±0.3	4.0±0.2	4.5±0.2	24.4±0.9	3.0±0.2	52.4±1.5
175	12.2±1.7	4.2±0.4	7.1±0.5	3.6±0.3	7.1±0.5	1.5±0.3	35.7±1.9
200	2.0±0.3	n.d	1.0±0.1	0.5±0.1	0.9±0.1	n.d	5.4±0.4

Glc-glucose; Gal-galactose; Man-mannose; Xyl-xylose; Ara-arabinose; Rha-rhamnoses in dependence of extraction temperature.

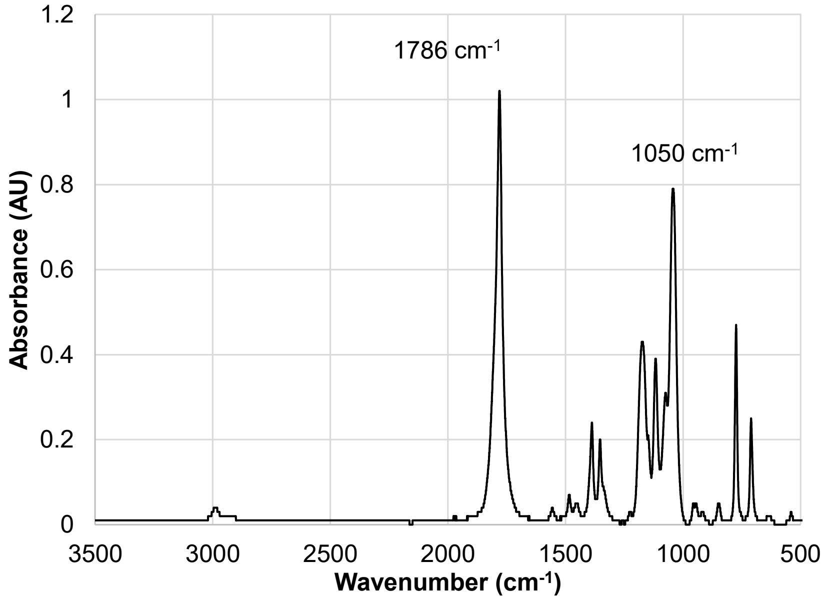

Figure S3. The FTIR spectra of PC

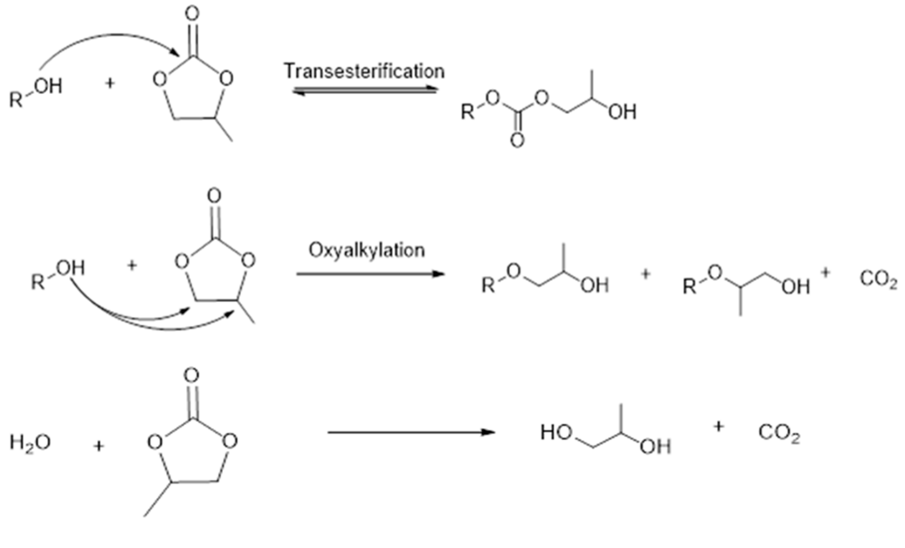

Figure S4. The pathways of the PC ring opening in its reaction with hydroxyl-containing compounds

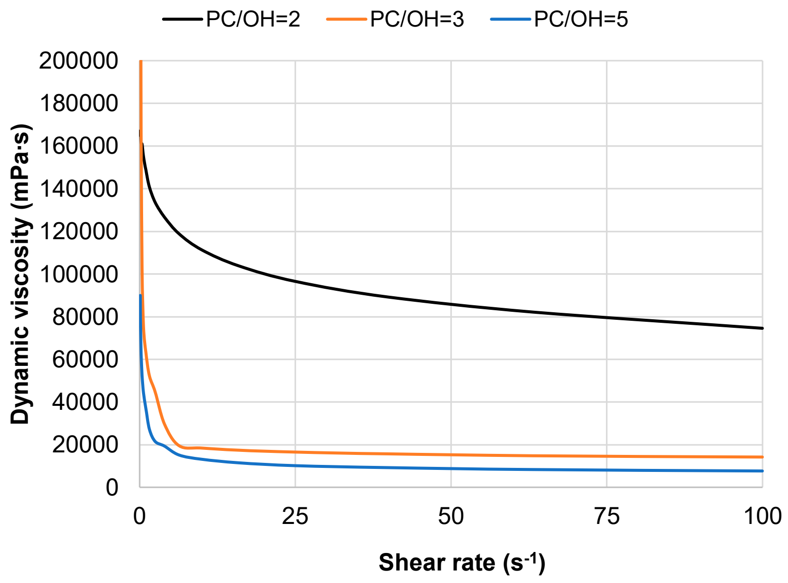

Figure S5. Share rate–viscosity curves of bio-polyol synthesized at different PC/OH ratios.

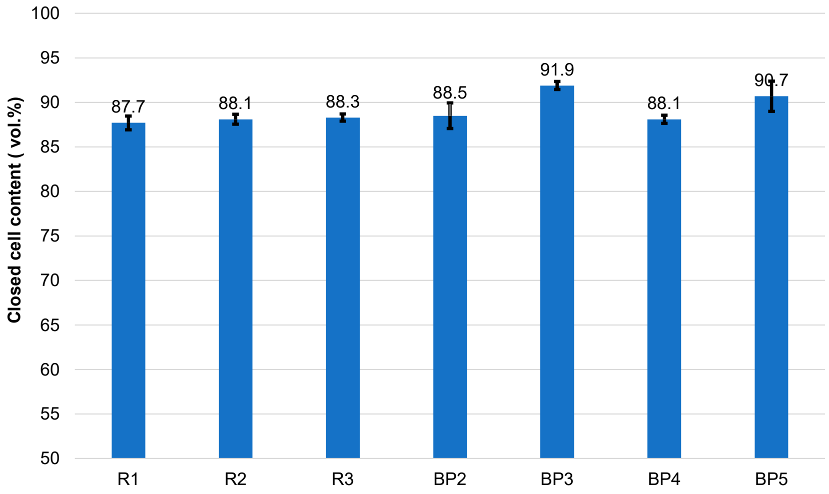

Figure S6. Closed-cell content by volume in PUR foams, references and with polyol systems substituted by 50% on bio-polyol (sample abbreviations are consistent with Table 4).

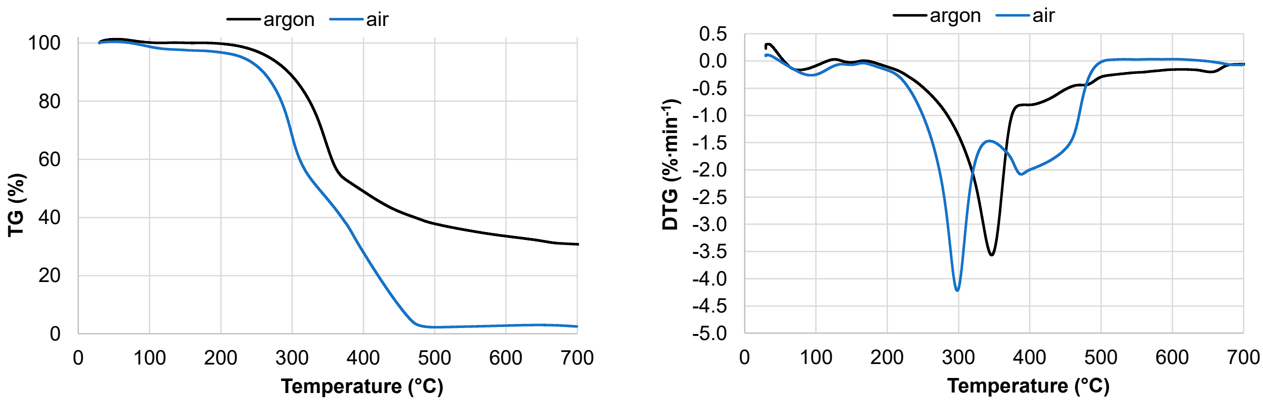

Figure S7. TG and DTG curves of extracted bark in argon and air.

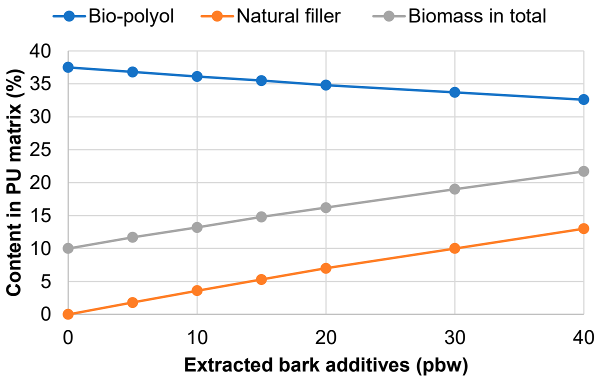

Figure S8. Content of bio-polyol, natural filler, and total biomass in the PU matrix as a function of extracted bark addition.

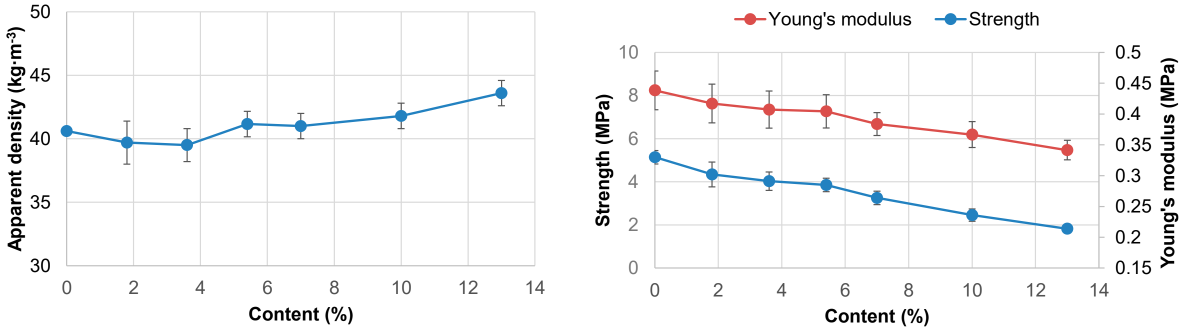

Figure S9. The effect of extracted bark content in the PU matrix on the apparent density of filled PUR foams and their mechanical characteristics under compression in the foaming direction.

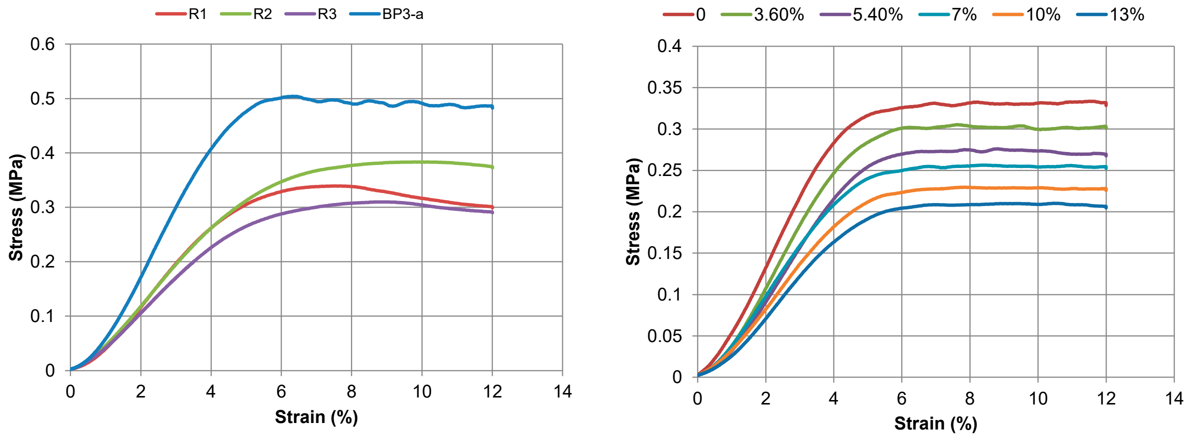

Figure S10 Stress–strain curves under compression for bio-polyol-based and reference PU foams (left) and the effect of extracted pine bark content in the PU matrix on the compression behavior of bio-polyol-based PUR foams (right) (sample abbreviations are consistent with Table 4 .and Table 5).
